# Cortical reorganization to improve dynamic balance control with error amplification feedback

**DOI:** 10.1186/s12984-022-00980-1

**Published:** 2022-01-16

**Authors:** Yi-Ching Chen, Yi-Ying Tsai, Gwo-Ching Chang, Ing-Shiou Hwang

**Affiliations:** 1 Department of Physical Therapy, College of Medical Science and Technology, Chung Shan Medical University, Taichung, Taiwan; 2 Physical Therapy Room, Chung Shan Medical University Hospital, Taichung, Taiwan; 3 Institute of Allied Health Sciences, College of Medicine, National Cheng Kung University, Tainan, Taiwan; 4 Department of Information Engineering, I-Shou University, Kaohsiung, Taiwan; 5 Department of Physical Therapy, College of Medicine, National Cheng Kung University, Tainan, Taiwan

**Keywords:** EEG, Error, Functional connectivity, Posture, Visual feedback

## Abstract

**Background:**

Error amplification (EA), virtually magnify task errors in visual feedback, is a potential neurocognitive approach to facilitate motor performance. With regional activities and inter-regional connectivity of electroencephalography (EEG), this study investigated underlying cortical mechanisms associated with improvement of postural balance using EA.

**Methods:**

Eighteen healthy young participants maintained postural stability on a stabilometer, guided by two visual feedbacks (error amplification (EA) vs. real error (RE)), while stabilometer plate movement and scalp EEG were recorded. Plate dynamics, including root mean square (RMS), sample entropy (SampEn), and mean frequency (MF) were used to characterize behavioral strategies. Regional cortical activity and inter-regional connectivity of EEG sub-bands were characterized to infer neural control with relative power and phase-lag index (PLI), respectively.

**Results:**

In contrast to RE, EA magnified the errors in the visual feedback to twice its size during stabilometer stance. The results showed that EA led to smaller RMS of postural fluctuations with greater SampEn and MF than RE did. Compared with RE, EA altered cortical organizations with greater regional powers in the mid-frontal cluster (theta, 4–7 Hz), occipital cluster (alpha, 8–12 Hz), and left temporal cluster (beta, 13–35 Hz). In terms of the phase-lag index of EEG between electrode pairs, EA significantly reduced long-range prefrontal-parietal and prefrontal-occipital connectivity of the alpha/beta bands, and the right tempo-parietal connectivity of the theta/alpha bands. Alternatively, EA augmented the fronto-centro-parietal connectivity of the theta/alpha bands, along with the right temporo-frontal and temporo-parietal connectivity of the beta band.

**Conclusion:**

EA alters postural strategies to improve stance stability on a stabilometer with visual feedback, attributable to enhanced error processing and attentional release for target localization. This study provides supporting neural correlates for the use of virtual reality with EA during balance training.

## Background

Accurate visual feedback can afford an additional benefit to self-monitoring that fosters sensorimotor integration and behavior success [1]. Contrary to the expectation that biased visual feedback could cause harmful perceptual conflicts [2], visual display of a worse performance outcome (or error amplification (EA) feedback) has recently been shown to improve task performance [3], possibly relating to perceptual narrowing or increase in attentional focus [4–6] according to the cue utilization hypothesis [7]. The functional benefits of using EA has been reported in a single-joint force task [5, 8], multiple-joint arm movement [9, 10], synergistic movements (such as postural balance [11] and human locomotion [12]. Also, EA has promising rehabilitative benefits to expedite motor recovery for neurological victims (Shirzad et al. 2016; [13]. According to the cue utilization hypothesis (Easterbrook et al. 1959), EA-related performance merits are attributable to perceptual narrowing, which facilitates richer and more frequent corrective attempts during motor task execution [4, 8, 14]. Nevertheless, how EA affects the neurocognitive control of human movements is not clear, though increase in task precision with EA have been validated at the behavioral level.

Postural control is not a completely automatic process under subcortical control. When the postural system is challenged with external perturbations [15–17], additional cortical resources are taxed to cope with balance constraints, leading to spectral changes in scalp-recorded electroencephalographic (EEG) signals over the motor, sensory, and cognitive regions [15, 18, 19]. An increase in local theta (4–7 Hz) connectivity of the frontal-central areas reflects detection of sensorimotor conflicts associated with falling risk [20, 21] and planning of corrective responses [22]. Decreases in alpha power (8–12 Hz) around the central, parietal and occipital regions may be related to increased attentional processes to monitor postural destabilization [23]. The availability of visual feedback (or visual inputs) has profound impacts on active postural control [22, 24, 25], for visual occlusion influences how the brain interprets balance contexts with fractional inadequacy of another sensory system (such as proprioception and vestibular inputs) [1, 26, 27]. The availability of visual feedback influences cortical adaptations to vibratory postural perturbations, with increases in the EEG spectral power of brain networks compared to that in closed-eye trials [22]. However, visual feedback of perturbation leads to beta (13–35 Hz) desynchronization in occipito-parietal areas, hypothetically relating to change in the postural strategy of the status quo [21, 28].

On account of the significance of visual referencing in balance control, the postural strategies are causally tuned to visual error amplification (EA) [29]. For a designated postural set, it was hypothesized that organization of the postural network (especially in the fronto-centro-parietal areas) using EA feedback should differ from that guided by traditional visual feedback. Challenge-based EA was expected to increase active participation of the brain for error processing, information mastery, and sensorimotor reintegration for enhanced error contexts. Considering the potential resource reallocation in the brain, this study investigated variations in regional activities and inter-regional connectivity of EEG sub-bands between EA feedback and real error (RE) feedback during stabilometer stance. The variations in EEG features should reflect EA-related modulation of postural strategies, as measured with postural dynamics at the behavioral level.

## Methods

### Participants

Eighteen young healthy adults (10 females and 8 males; age: 24.1 ± 2.2 years) from a university campus participated in this study. This study was approved by an authorized institutional human research review board at the University Hospital (No. B-ER-105-032). Prior to the experiment, all subjects read and signed personal consent forms, in accordance with the Declaration of Helsinki.

### Experimental procedures and instrumentation

**Fig. 1:**
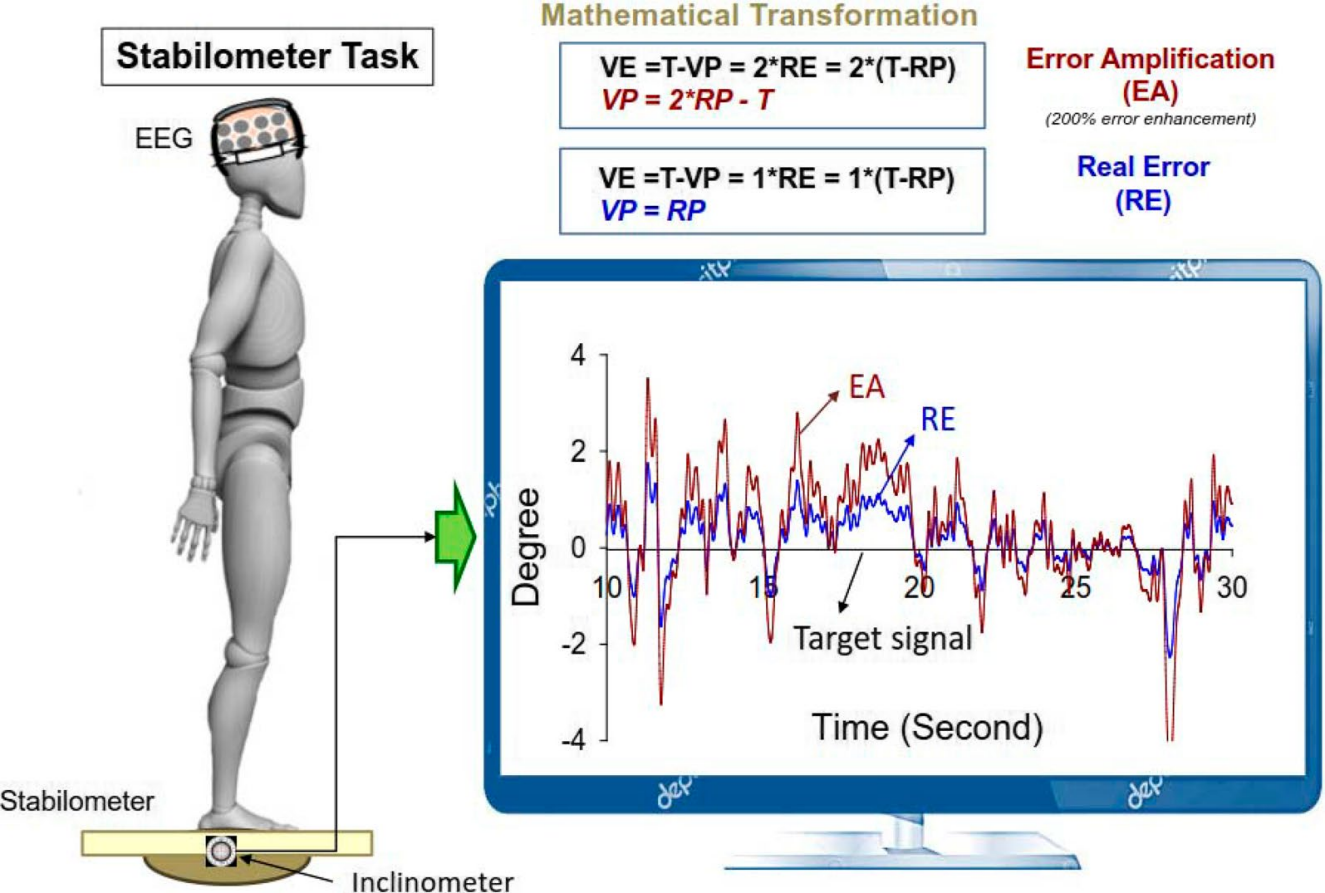
System setup and error augmentation feedback. With mathematical transformation, the visualized postural sway in the error amplification (EA) condition virtually doubled the size of the real execution error displayed in the real error (RE) conditions. The mathematical transformation is displayed in the formula boxes. In contrast, the subjects in the RE condition received on-line error feedback without any manipulation. The subjects could either see real feedback or error amplification feedback, depending on their grouping. (VP: visualized postural sway; RP: real postural sway; VE: visualized error; RE: real error; T: target signal)

This study used a randomized, repeated measures design. The subjects in this study were requested to stand on a 50 × 58 cm stabilometer (radius: 25 cm; height: 18.5 cm) and maintain a steady stance with two forms of visual feedback (real error (RE) and error amplification (EA)) (Fig. 1). The subjects were visually guided to maintain the steady stabilometer stance. Both the trajectory of stabilometer plate movement and horizontal target line that represented the ground level were displayed in the visual feedback. The subjects needed to meticulously couple the trajectory of plate movement to the horizontal target line during stabilometer stance. In the RE condition, the visualized postural sway (VP) was identical to the trajectory of stabilometer plate movement, and the visualized error was equal to the real error (VE = RE)( Fig. 1). In the EA condition, the VP was not the real trajectory of stabilometer plate movement. Instead, the VP was mathematically transformed with VP = 2*RP-T (RP: real postural sway, T: target signal). Hence, the size of the visualized error (VE) in the visual feedback was twice that of the real error (VE = 2*RE) [4, 5, 8]. Namely, the participants perceived execution errors that were virtually doubled in the EA condition. The participants were not informed of the kind of visual feedback during the experiment. There were three 60 s experimental trials for the RE and EA conditions, which were randomly assigned. A 3 min pause were interleaved between two experimental trials to prevent a sense of fatigue.

The tilting angle of the plate movement was registered with an inclinometer (Model FAS-A, LORD MicroStrain, USA) mounted on the center of the stabilometer. The EEG dataset was recorded continuously using a NuAmps amplifier (NeuroScan Inc., EI Paso, USA) and Ag–AgCl scalp electrodes, according to the International 10–20 system. Scalp EEG signals were localized at different cortical areas (Fp_1/2_, F_z_, F_3/4_, F_7/8_, FT_7/8_, FC_z_, FC_3/4_, C_z_, C_3/4_, CP_z_, CP_3/4_, P_z_, P_3/4_, T_3/4_, T_5/6_, TP_7/8_, O_z_, and O_1/2_) [15, 30]. Reference electrodes were placed on each side of the mastoid process (A_1_/A_2_), and the ground electrode was placed on the forehead. For subtraction of eye movement and blink artifacts, horizontal electrooculography (EOG) data were collected with two electrodes placed at the outer canthus of the left and right eyes. For off-line vertical EOG assessment, two electrodes were placed infra- and supra-orbitally at the right eye, respectively. The impedances of all the electrodes were below 5 kΩ and were referenced to linked mastoids of both sides. The EEG data were recorded by setting a band-pass filter (cut-off frequencies: 0.1–70 Hz) and a 60 Hz notch filter. The EEG data and the angular plate movement were integrated and synchronized by the AD controller of the LabView platform (Labview v.8.5, National Instruments, USA). The sampling rate was set at 1 kHz.

### Data analysis

#### Characterization of postural fluctuations

Data were analyzed off-line in Matlab R2019a (The Mathworks Inc., Natick, MA, USA). The angular plate movements were first conditioned with a zero-phase low-pass filter (cut-off frequency: 4 Hz), followed by linear transformation to degrees of the postural task. The first 2 s and the last 2 s of the sampled data of each trial were automatically discarded to avoid unrepresentative data in case the participants were not fully stable at the beginning and end. The size and regularity of postural fluctuations were represented with the root mean square (RMS) [30] and sample entropy (SampEn) [31, 32] of the angular plate movements after removal of a linear trend, respectively. To preclude the amplitude effect on SampEn, SampEn was calculated with standardized angular plate movements, or normalization of the postural fluctuations with the standard deviation of the time series. SampEn ranges from 0 to 2, with the larger value representing more complexity [33]. SampEn measured the negative natural logarithm of an estimate of the conditional probability that epochs of length *m* that matched point-wise within a tolerance level (*r*) also matched at the next point for time series data with a total data point number of N. The mathematical formula for SampEn was $SampEn\left(m,r,N\right)=-\mathrm{log}\left(\frac{{\sum }_{i=1}^{N-m}Ai}{\sum_{i=1}^{N-M}Bi}\right)$, where *r* = 15% of the standard deviation of the error signal and *m* = 2. Spectral profiles of postural fluctuations were estimated with a fast Fourier transform and the Welch method (Hanning window, window length: 30 s, 20% overlapping segment) with a spectral resolution of 0.01 Hz. The mean frequency (MF) of postural fluctuations was calculated with the spectra of postural fluctuations.

#### Regional activities and inter-regional connectivity of EEG sub-bands

**Fig. 2:**
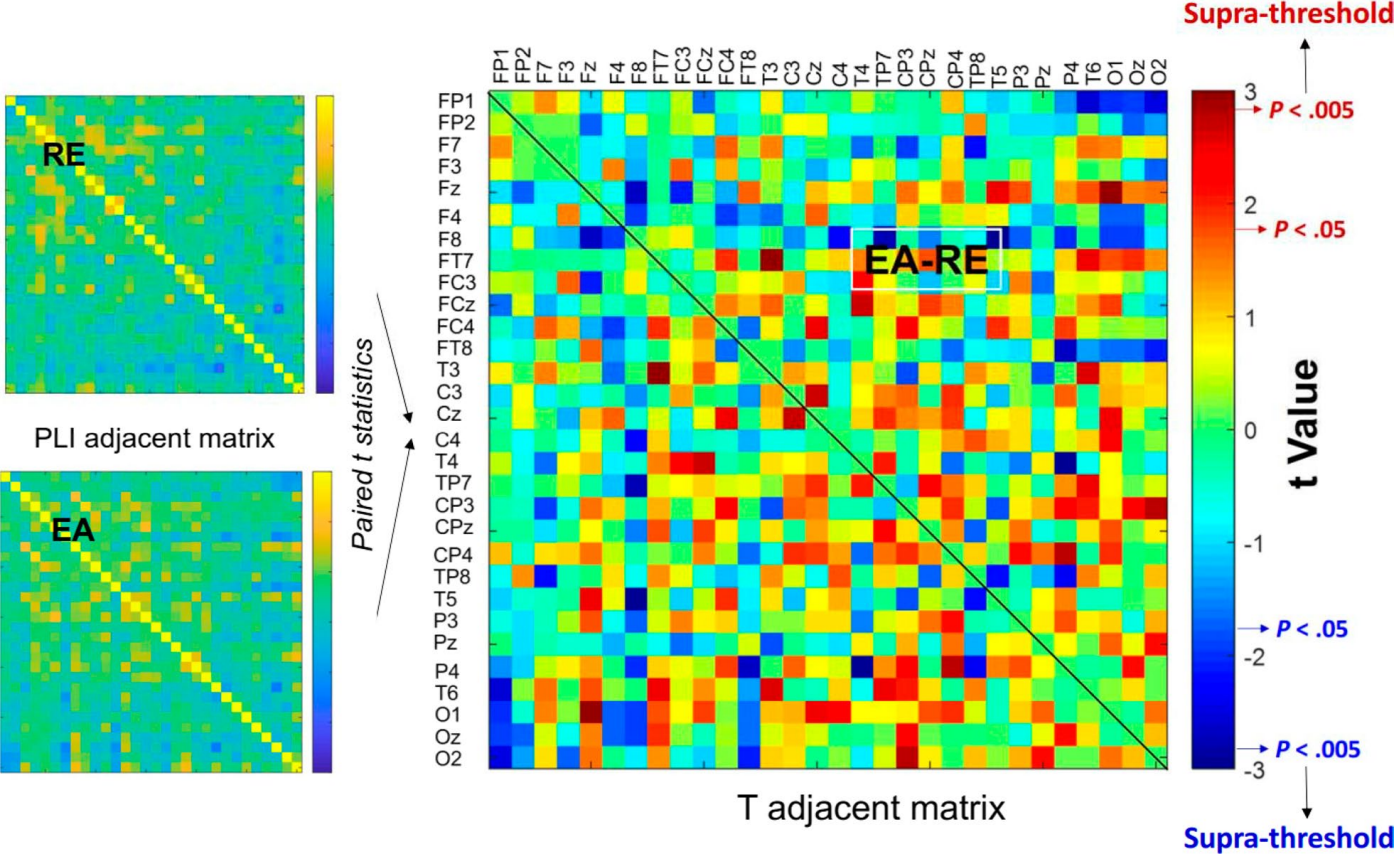
Schematic illustration of the contrast difference in sub-band inter-regional connectivity between the EA and RE conditions. The red color indicates a greater inter-regional connectivity in the EA condition than that in the RE condition. The blue color indicates a smaller inter-regional connectivity in the EA condition than that in the RE condition. The supra-threshold connectivity is defined as high level of significant differences in functional connectivity between the EA and RE conditions. Only supra-threshold connectivity is further considered for network-based analysis. (EA: error amplification, RE: real error)

All the EEG data were first conditioned with a band-pass filter (cut-off frequencies: 1 and 60 Hz) using a zero-phase finite impulse response (FIR) filter (60 dB/octave). The blinks were detected by creating a bipolar vertical EOG channel by subtracting activity in the infraorbitally-placed electrode from that in the superorbitally-placed electrode. Correction of ocular artifacts was performed with the NeuroScan 4.3 software program (NeuroScan Inc., EI Paso, TX, USA). As with the stabilometer plate data, the EEG signals of the first and last 2 s were excluded from analysis. The remaining EEG data were segmented into 2-s epochs and visually inspected for undetected artifacts by the researchers. Regional cortical activity was characterized with relative power for each EEG channel, based on the fast Fourier transform in the following spectral ranges: theta (4–7 Hz), alpha (8–12 Hz), and beta (13–30 Hz). Oscillations under 4 Hz were not analyzed because they are susceptible to vigorous movement artifacts. Also, we did not analyze EEG data in the gamma band, because they are more easily contaminated by muscle activity. Based on the normalized power spectra of an epoch, the mean peak amplitude in each sub-band was obtained across the EEG channels. All the spectral measures of the three trials were averaged in the EA and RE conditions. The regions of interest depended on the EEG channels where the sub-band peak amplitude was most evident in the scalp during the stabilometer stance. For instance, theta and alpha powers were most prominent in the mid-frontal (F_z_ and FC_z_) and occipital areas (O_1_, O_2_, and O_z_). Pronounced beta power was noted at the left (T_3_, TP_7_, and T_5_) and right (T_4_, TP_8_, and T_6_) temporal areas, in addition to the prefrontal area (FP_1_ and FP_2_). In addition, phase-lag index (PLI) was used to index the inter-regional connectivity of the 30 electrode pairs from all sub-bands. The PLI described the distribution asymmetry of phase differences in the instantaneous phases of two time-series based on the Hilbert transformation. If $\varphi (t)$ is the phase difference, the PLI is defined thus:$PLI=\left|E\left\{sgn(\Delta \varphi (t))\right\}\right|$, where *sgn* is a function that extracts the sign of a real number. PLI was used because it is relatively immune to volume conduction [34]. Computation of the PLI across all electrode pairs resulted in a square 30 × 30 PLI adjacent matrix for the RE and EA conditions (Fig. 2). The PLI-based functional connectivity was calculated with the HERMES function in Matlab [35].

### Statistical analysis

EEG variables and postural variable in the three experimental trials in the EA and RE conditions were averaged for each subject. Multi-variate Hotelling's T-squared statistics were used to examine the feedback effect (EA vs. RE) on the postural fluctuation characteristics (RMS, MF, and SampEn). The post-hoc test was independent t test with the level of significant difference identical to the Simes test. The Simes test would not produce over-correction, unlike the Bonferroni test. For all post-hoc hypotheses ($H={\cap }_{i=1}^{m}$), the Simes test did not reject elementary *H*_*i*_ if *p*_*i*_ ≤ i*0.05/m for ordered unadjusted p values (*p*_*1*_ ≤ … ≤ *p*_*m*_). The type 1 error rate using the Simes test proved to be exactly 0.05. Paired t test was used to contrast differences in the pooled sub-band powers of the EEG channels in the regions of interest between the EA and RE conditions (Fig. 2). The connectivity strength within the PLI adjacent matrix in the EA and RE conditions was contrasted with paired t tests. A set of supra-threshold connections ($\left|{t}_{17}\right|$> 2.898, *p* < 0.005) was extracted to highlight the differences in topological distributions between the EO and EC conditions (Fig. 2). Only the practice-related differences in the functional connectivity (or inter-regional PLI values) that exceeded an uncorrected threshold (*p* = 0.005) were defined as a set of supra-threshold connectivity. To examine feedback-dependent variations in the network-based supra-threshold connectivity, a permutation test was performed 10,000 times to examine variations in the null distribution of the supra-threshold connectivity between the RE and EA conditions. Methodological details of corrected network-based statistics were documented in the work of Zalesky et al. [36]. Data are presented as group means ± standard deviation. All statistical analyses were performed in IBM SPSS Statistics (v19). Based on the averaged EEG variables and postural sway variables from the three experimental trials for each participant (n = 18), Pearson’s correlation was performed to examine the significance of correlations between normalized differences in postural sway metrics between the EA and RE conditions ((EA-RE)/RE) and variations in sub-band regional activities/inter-regional connectivity that were dependent on feedback mode. The level of significance was 0.05.

## Results

Table 1 shows the results of Hotelling's T-squared statistics for contrasting the characteristics of postural fluctuations between the EA and RE conditions during stabilometer stance. The postural fluctuation characteristics varied significantly with feedback patterns (Wilks' Λ = 0.478, *p* = 0.010). Post-hoc analysis indicated that EA exhibited smaller RMS (*p* = 0.005), greater SampEn (*p* = 0.009), and higher MF (*p* = 0.015) of postural fluctuations than RE did.

**Table 1:** The contrast angular fluctuation dynamics of plate movement between stabilometer stance with the real error (RE) and error amplification (EA) feedbacks

	RMS (deg)	SampEn	MF (Hz)	Statistics
RE	1.125 ± .126	.197 ± .019	.342 ± .023	Λ = 0.478, *p* = .010
EA	.808 ± .122^††^	.260 ± .028^*^	.410 ± .031^*^	RMS: *t*_*17*_ = -3.243, *p* = .005, SampEn: *t*_*17*_ = 2.972, *p* = .009 MF: *t*_*17*_ = 2.708, *p* = .015

(*RMS* root mean square of angular fluctuations, *MF* mean frequency of angular fluctuations, *SampEn* sample entropy of angular fluctuations) (††: EA < RE, p .005; *: EA > RE, p < .05)

**Fig. 3:**
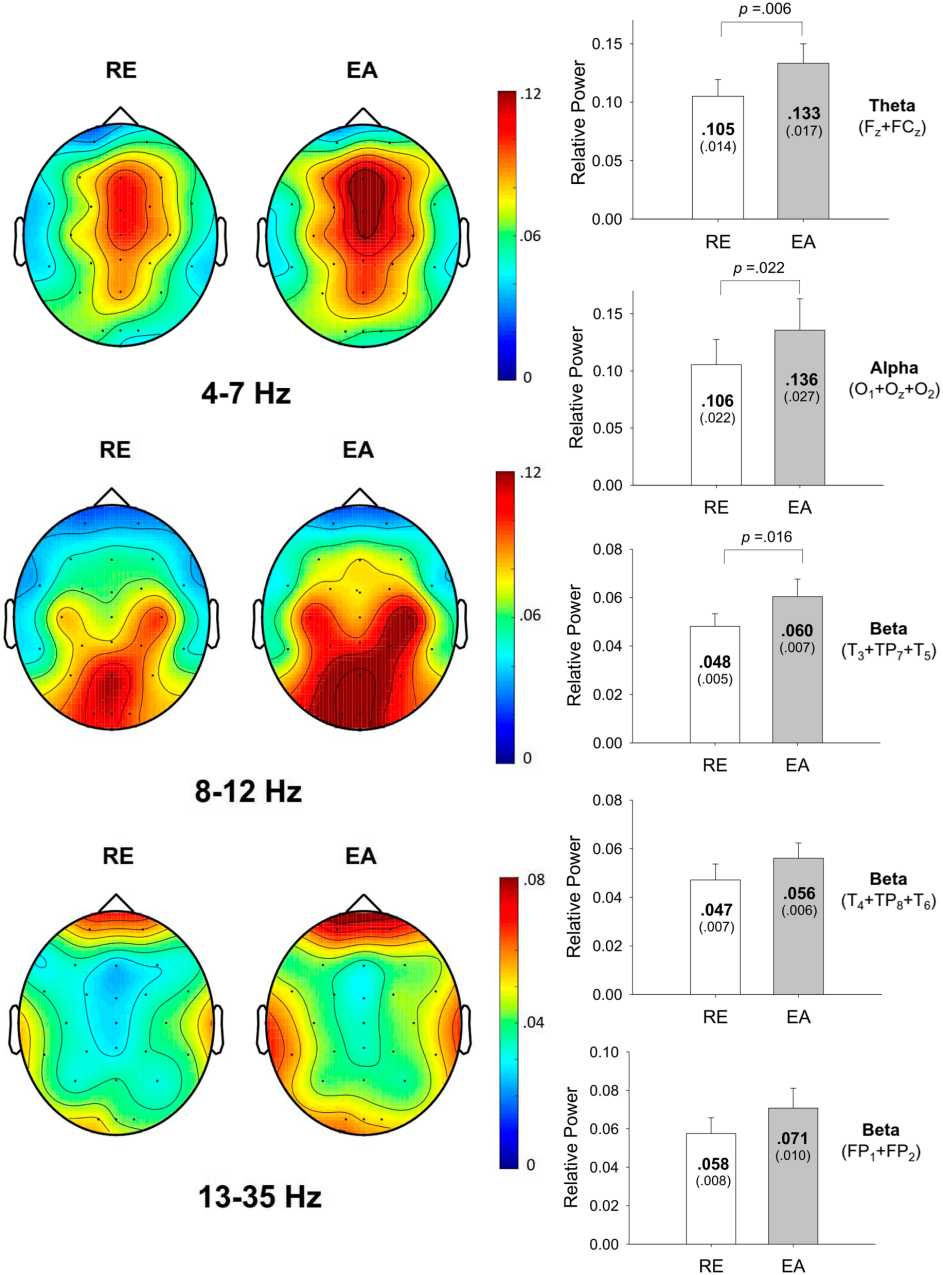
The pooled topological spectral mapping of scalp EEG at theta (4–7 Hz), alpha (8–12 Hz) and beta bands (13–35 Hz) in the EA and RE conditions. Oscillatory waves in the theta and alpha bands are most evident in the mid-frontal (F_z_ and FC_z_) and occipital (O_1_, O_2_, and O_z_) areas, respectively. Beta oscillation is most evident in the left temporal (T_3_, TP_7_, and T_5_), right temporal (T_4_, TP_8_, and T_6_) and prefrontal (FP_1_ and FP_2_) areas. The mean relative power of each sub-band in the target regions is contrasted between the EA and RE conditions. There were significant EA-induced differences in pooled mid-fontal theta, pooled occipital alpha, and pooled left temporal beta oscillations (*p* < .05). (EA: error amplification; RE: real error)

Figure 3 shows the topological distribution of the relative powers of the theta, alpha, and beta rhythms during stabilometer stance with RE and EA. It was evident that the theta and alpha powers were the most evident in the mid-frontal and occipital areas, respectively. Pronounced beta power was noted in the bilateral temporal areas and prefrontal area. The results of paired t test showed that the pooled relative power of the mid-frontal theta oscillation (F_z_ and FC_z_) was greater in the EA condition than in the RE condition (*t*_*15*_ = -3.126, *p* = 0.006)(Fig. 3, right plot). The pooled relative power of occipital alpha oscillation (O_1_, O_2_, and O_z_) was greater in the EA condition than in the RE condition (*t*_*15*_ = -2.512, *p* = 0.022). Only the left temporal beta oscillation (T_3_, TP_7_, and T_5_) was significantly tuned to a feedback mode with greater pooled beta relative power in the EA condition (*t*_*15*_ = − 2.664, *p* = 0.016). Despite an increasing trend, the pooled relative beta powers of the right temporal (T_4_, TP_8_, and T_6_)(*t*_*15*_ = -1.555, *p* = 0.138) and prefrontal (FP_1_ and FP_2_)(*t*_*15*_ = -1.836, *p* = 0.084) areas were not significantly different between the two feedback modes.

**Fig. 4:**
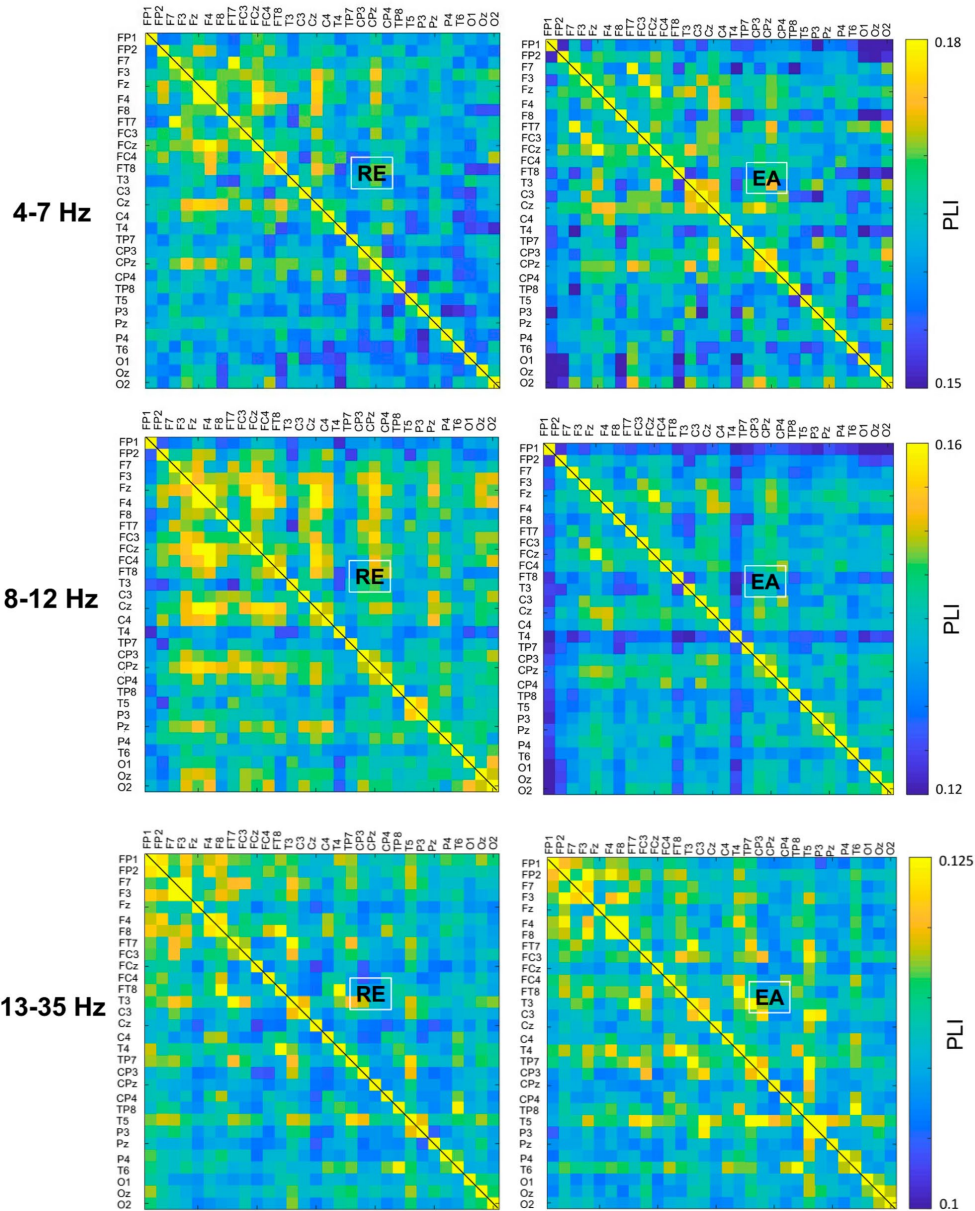
The PLI adjacent matrices that represent the averaged strength of inter-regional connectivity of the theta (4–7 Hz), alpha (8–12 Hz), and beta (13–35 Hz) bands between the EA and RE conditions. (*PLI* phase-lag index, *EA* error amplification, *RE* real error)

**Fig. 5:**
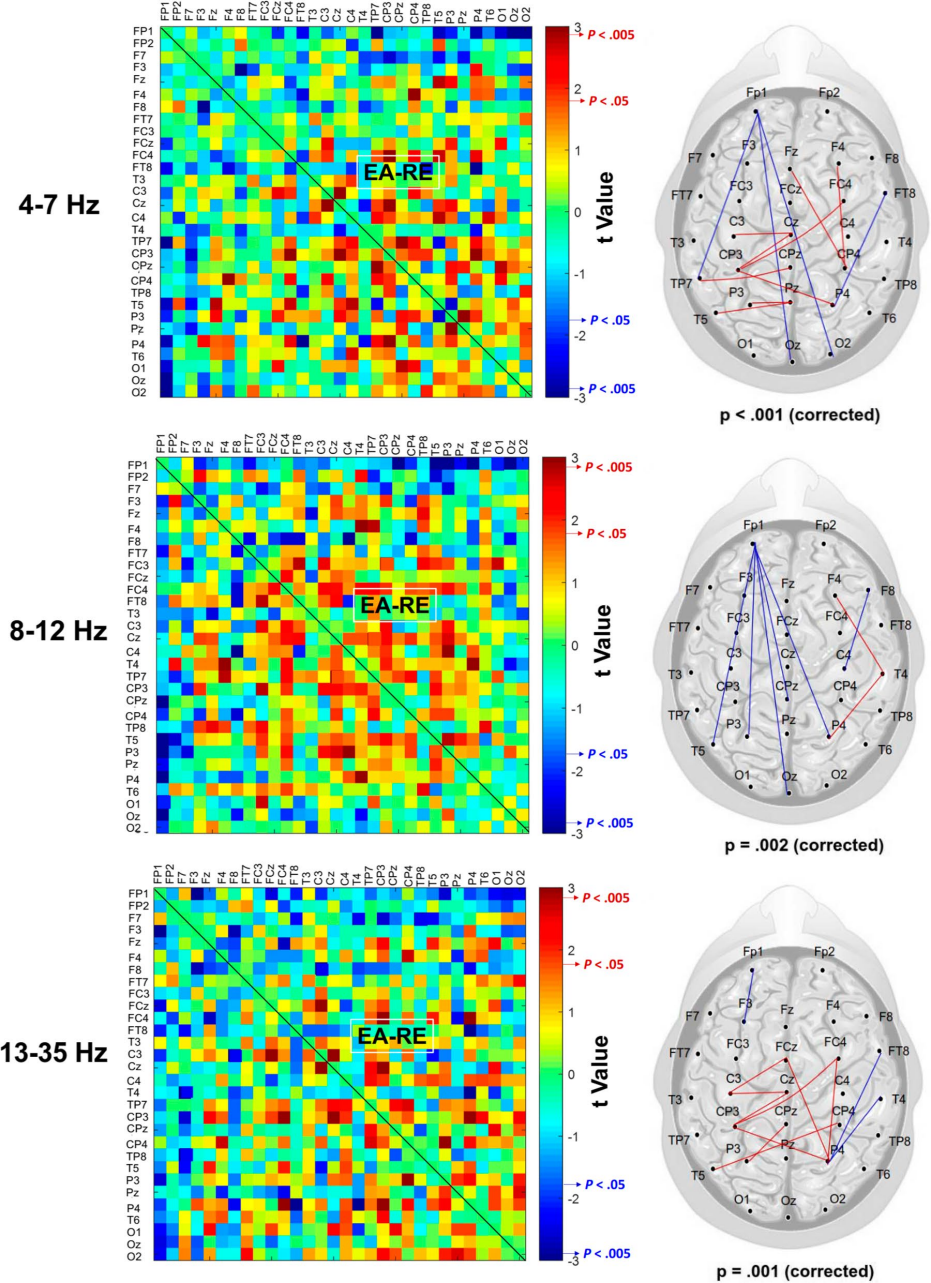
The adjacent matrices of t values that contrast PLI values of all electrode pairs between the EA and RE conditions. A contrasting wiring diagram on the scalp shows the topological distributions of the supra-threshold connectivity tuned to error amplification (EA vs. RE)(|*t* |> 2.898, *p* < .005). It is worth noting that EA results in reductions of the prefrontal-parietal/occipital connectivity (alpha/beta bands) and right tempo-parietal connectivity (theta/alpha bands). Meanwhile, EA also enhances fronto-centro-parietal connectivity (theta/alpha bands) and right temporo-frontal/temporo-parietal connectivity (beta band). (Red line: EA supra-threshold connectivity > RE supra-threshold connectivity, *p* < .005; Blue line: RE supra-threshold connectivity > EA supra-threshold connectivity, *p* < .005). (*PLI* phase-lag index, *EA* error amplification, *RE* real error)

Figure 4 shows the pooled PLI adjacent matrix of theta, alpha and beta oscillations during the stabilometer stance with the EA and RE. The differences in PLI for all electrode pairs of the sub-sub-bands between the EA and RE were represented with the adjacent matrix of t-values (Fig. 5, left plots). According to the adjacent matrix of t-values, the supra-threshold connectivity of theta, alpha and beta bands was labeled in the scalp map ($\left|{t}_{17}\right|$> 2.898, *p* < 0.005)(Fig. 5, right plots). The results of corrected network-based statistics indicated that the feedback configuration differentially modulated the brain functional connectivity during stabilometer stance (theta (*p* = 0.001, corrected), alpha (*p* < 0.001, corrected), and beta (*p* = 0.002, corrected)). For the theta band, EA mainly resulted in global increases in fronto-centro-parietal connectivity and decreases in functional connectivity between 1) the left prefrontal (FP_1_) and left frontal (F_3_) areas, 2) the right temporal (T_4_ and FT_8_) and parietal (P_4_) areas (Fig. 5, upper right plot). For the alpha band, long-range functional connectivity between FP_1_ and left temporal-parietal (TP_7_)/occipital (O_z_ and O_2_) areas waned with the use of EA (Fig. 5, middle right plot). Moreover, EA resulted in enhanced fronto-centro-parietal alpha connectivity. For the beta band, EA decreased the long-range functional connectivity between the left prefrontal (FP_1_) and left temporal (T_5_)/centro-parietal-occipital (P_3_, CP_z_, P_4_, and O_z_) areas (Fig. 5, lower right plot). In contrast, the functional connectivity of the right temporal area (T_4_) and right frontal (F_4_)/parietal (P_4_) areas was potentiated with EA.

Table 2 summarizes the Pearson’s correlations between feedback-dependent regional activity and postural sway dynamics. There was a significant correlation between EA-dependent differences in the pooled mid-frontal theta rhythm (△ Mid-frontal theta (F_z_ + FC_z_)) and normalized differences in the RMS of postural fluctuations (*r* = -0.488, *p* = 0.040). Marginally significant correlations were noted in 1) the pooled occipital alpha rhythm (△ Occipital alpha (O_1_ + O_2_ + O_z_)) and normalized differences in the RMS of postural fluctuations (*r* = -0.467*, p* = 0.051), and 2) the pooled mid-frontal theta rhythm (△ Mid-frontal theta (F_z_ + FC_z_)) and normalized differences in MF of postural fluctuations (*r* = 0.428*, p* = 0.077). However, none of the EA-dependent changes in the inter-regional connectivity of all sub-bands were significantly correlated to normalized changes in sample entropy of postural fluctuations (ND-SampEn)(*p* > 0.05).

**Table 2:** Pearson’s correlation between feedback-dependent regional activity and postural sway dynamics

Pearson’s correlation* (n* = *18)*	ND-RMS	ND-SampEn	ND-MF
△ Mid-frontal Theta (F_z_ + FC_z_)	***r* = **− **.488, *p* = .040**	*r* = − .069, *p* = .785	*r = .428, p = .077*
△ Occipital Alpha (O_1_ + O_2_ + O_z_)	*r = − .467, p = .051*	*r* = − .061, *p* = .806	*r* = .371, *p* = .131
△ Left Temporal Beta (T_3_ + TP_7_ + T_5_)	*r* = .157, *p* = .533	*r* = − .310, *p* = .210	*r* = − .014, *p* = .955

The black-bolded and italic fonts indicate significant and marginally significant correlations of regional activity and postural sway dynamics, respectively. (*ND* normalized differences, *RMS* root mean square of angular fluctuations, *MF* mean frequency of angular fluctuations, *SampEn* sample entropy of angular fluctuations)

## Discussion

At the behavioral level, EA added to postural stability in stabilometer stance with a smaller size of postural fluctuations as compared to the RE condition (or traditional visual feedback). The EA-related decline in postural sway corresponded with selective modulations of sub-band regional activities and inter-regional connectivity of the EEG signals. Compared to traditional visual feedback, EA resulted in greater mid-frontal theta, occipital alpha, and left temporal beta oscillations during stabilometer stance. In addition, EA potentiated the fronto-centro-parietal connectivity of the theta/alpha bands and the right temporo-frontal and temporo-parietal connectivity of the beta band. In addition to the right tempo-parietal connectivity of the theta band, the long-range prefrontal-parietal and prefrontal-occipital connectivity of the alpha and beta bands was downward modulated with EA during stabilometer stance.

### Characteristic changes of postural fluctuations with EA

In view of the smaller size of postural fluctuations (Table 1), EA-related reduction in dynamic postural sway on a stabilometer was consistent with previous observations on other timing-based motor tasks, such as arm reaching [37], postural shifting [38], force tracking [4, 8, 14] and locomotion [12]. The augmented errors underscored tracking deviations associated with changes in postural fluctuation dynamics with greater sample entropy and higher mean frequency (Table 1). In the framework of intermittent motor control [39], postural fluctuations originate from superimposition of a number of pulse elements centrally scaled to adjust movement deviations toward the target goal. In support of postural fluctuations of higher frequency and greater complexity, the postural system with EA tended to maintain the target position in more frequent and elaborate corrective attempts, similar to the observations from force-tracking tasks [4, 8, 14].

### Modulations of sub-band regional activities with EA

Originating from the anterior cingulate cortex, the frontal midline theta has been proposed to participate in error elaboration to optimize motor [40] and cognitive [41] tasks. The role of synchronized large-amplitude low-frequency theta oscillation is to entrain cortical activities across disparate areas, thereby focusing on a narrower subset of integrated information to reduce errors. Hence, greater mid-frontal theta waves with EA might serve to elaborate the most-likely likelihood from error contexts with emphasis on worse outcomes (Fig. 3), though they were virtually faked. Previous studies considered that the theta power over frontal electrodes emerged for increasing needs to cope with postural destabilization when balance tasks became more difficult [23, 42], such as during unipedal stance or sudden postural perturbations [17, 19, 43]. In this study, on account of significant negative correlation between ND-RMS of postural fluctuation and △ Mid-frontal Theta (F_z_ + FC_z_) (Table 2), smaller postural sway during stabilometer stance with EA were associated with greater EA-induced increases in pooled mid-frontal theta rhythm. We might well attribute the performance benefits of EA on stabilometer stance to a neurocognitive process, in favor of reinforced error processing to minimize faked error augmentation. To a certain extent, EA-evoked theta augmentation could also modulate postural adjustments contingent on uncertainty-driven exploration [44]. For the use of EA, the participants with higher EA-induced change in pooled mid-frontal theta rhythm tended to increase the rate of postural corrections, in view of the marginally significant positive correlation between and △ Mid-frontal Theta (F_z_ + FC_z_) and ND-MF of postural fluctuations (*p* = 0.077) (Table 2). The neural evidence supporting a corrective role of the mid-frontal theta oscillations was also observed in visuomotor tracking [45], during which submovements used to remedy tracking deviations were time-locked to 3–7 Hz scalp EEG.

Oscillatory alpha-band (8–12 Hz) activity, the most prominent features of the EEG in the posterior brain, is tuned to both the availability of visual input [24] and the posture load [18]. Occlusion of visual inputs resulted in higher levels of alpha activity at the parietal-occipital areas, in agreement with the prediction of the cortical idling hypothesis, which posited neuronal processing of task-irrelevant inhibition with alpha oscillations (Pfurtscheller et al. 1996). In contrast, increases in balance task difficulty resulted in a decline in fronto-parietal alpha activities [18, 23], possibly reflecting increased attentional processes [18, 42], or parallax-sensitive remapping [46] to regulate postural responses. Notably, the occipital alpha power during stabilometer stance was also enhanced with EA in this study (Fig. 3). The ensuing alpha augmentation would suggest that less visuospatial attention was paid to precisely represent the visual space of the target and performance trajectory during postural tracking, granting that detection of salient tracking mismatches became easier with EA. The argument of neural economy based on alpha idling gained empirical support from the marginally significant correlation between increases in occipital alpha power and decreases in the RMS of postural fluctuations for the application of EA (Table 2). Oscillatory beta-band (13–35 Hz) activity in the left temporal area was also magnified with EA (Fig. 3). One of the important functions of the left temporal lobe (T3, T5) is to recognize visual perception of what an object is and to provide a verbal mode of visual information processing [47]. Cortical beta activity in the temporal and occipital areas has been proposed to link with sensory gating [48, 49]. Changes in beta power have also been reported during movements that create incongruence between visual and somatosensory/proprioceptive inputs [48]. However, the functional implications for changes in the left temporal beta activity with EA are not exactly clear. Perhaps the beta enhancement reflected perceptual conflicts between the visual and non-visual sensory modalities, as only the visual input was biased. The adverse effect was not influential in affecting postural control, considering the lack of a significant correlation between variations in the regional beta enhancement and postural fluctuation dynamics with EA (Table 2).

### Rewiring of sub-band functional connectivity with EA

EA resulted in selective modulations of functional connectivity in postural networks (Fig. 5). One of the most prominent changes was decreases in the long-range connectivity between the left prefrontal (FP_1_) and the temporal/parietal/occipital (TP_7_, P_3_, P_4_, CP_z_, T_5_, O_2_, or O_z_) regions in the alpha or beta band (Fig. 5, the middle and bottom rows). The frontal-eye field, which receives direct connections from extrastriate visual areas, sends projections to oculomotor structures to pursue visual targets with eye movements [50, 51]. The dorsolateral prefrontal cortex and other cortical structures (parietal lobe and temporo-occipital lobe) are orchestrated to form the fronto-parieto-occipital network, which gates spatial attention for visual discrimination during a complex visual search task [52, 53]. For a unimanual visuomotor task requiring integration of a pinch act with visual feedback, the fronto-parieto-occipital network, bridged by alpha oscillations, was considered to integrate instructed motor responses with feature information elaborated in the dorsal stream [54]. In addition to the prefrontal-parietal and prefrontal-occipital connectivity of alpha and beta bands, EA also undermined the connectivity strength in the right temporo-parietal connectivity (P_4_–FT_8_ and P_4_–T_4_) at the theta and alpha bands (Fig. 5, upper and middle rows), which coordinates the middle and inferior frontal gyri to reorient spatial attention for detecting behaviorally relevant visual stimuli [55, 55],[56]. Stroke patients with damage to this connectivity exhibit atypical reductions in EEG theta coherence [57] and the syndrome of neglect [58]. In conjunction with the above-mentioned visual idling with EA for enhanced occipital alpha activity, decreases in the neural synchronization among these distinct areas with EA imply less top-down visual awareness for target detection during the visually-guided stabilometer stance. Considering the concurrent enhanced mid-frontal theta, an integrative explanation could be that active inference of EA highlighted implicit error processing [59] such as fast error evaluation (Chernyshev et al. 2017) and response selection [60], rather than explicit detection of the salient tracking mismatches during the stabilometer stance.

**Fig. 6:**
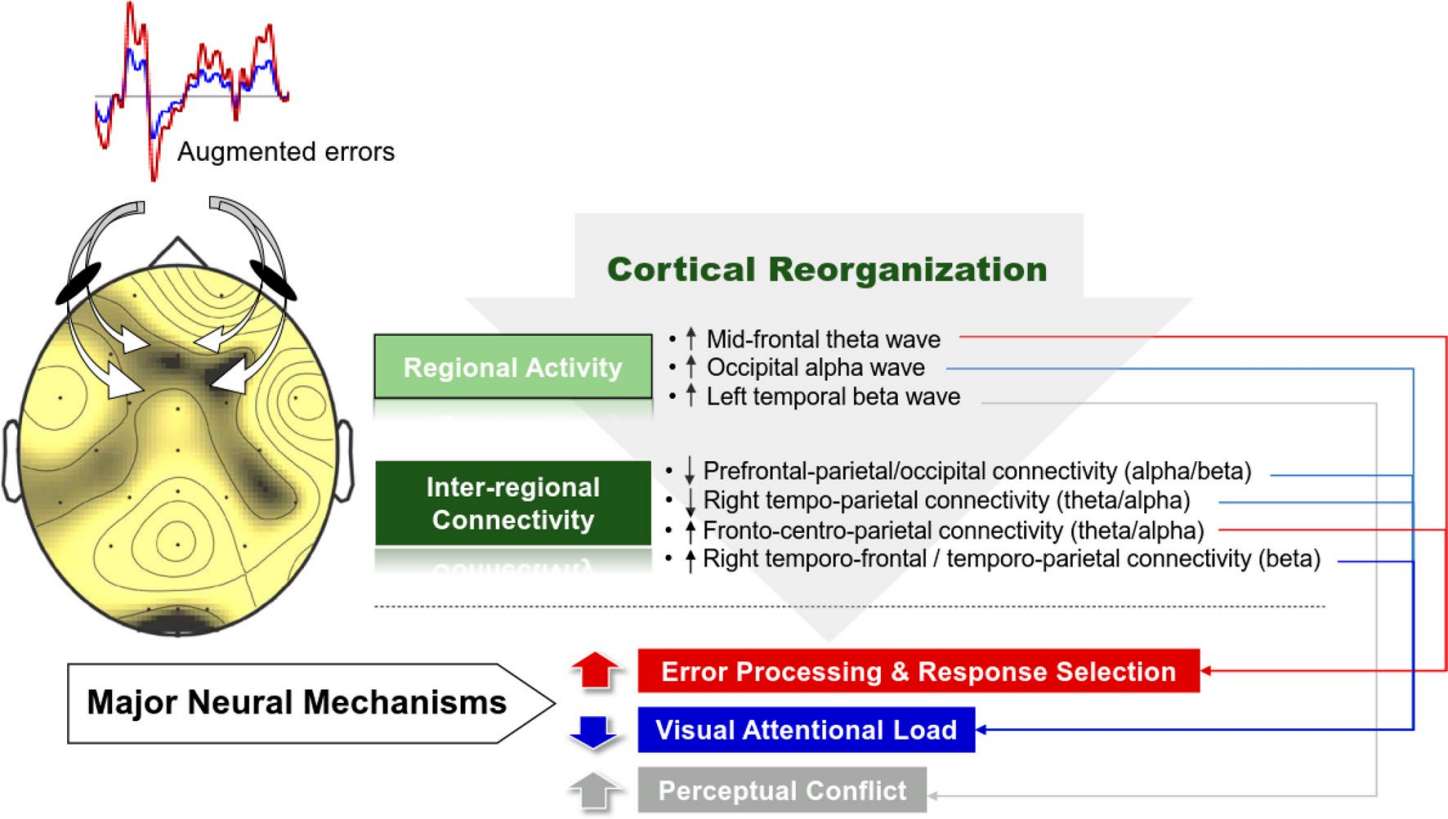
Hypothesized cortical mechanisms underlying enhanced postural control with error amplification feedback

At the theta and alpha bands, functional connectivity within the posterior somatosensory association area and premotor/motor cortex, critical to sensorimotor integration, was conversely potentiated with EA. An increase in theta activity of the network is a neural signature of selecting the alternate responses after incongruence of action and cue information [61]. For a postural task, the theta activity of the network was enhanced when postural balance was challenged with a narrow base of support and an unstable surface [42, 62]. In addition, increment of the balance task difficulty corresponded with a decline in alpha coherence across the centro-parietal region [18], due to an increase in the state of outcome uncertainty [63]. The source of the alpha connectivity was hypothesized to be the occipital lobe, which propagates to the centro-parietal areas via the magnocellular and parvocellular systems [62]. Despite sham difficulty and uncertainty fabricated by EA, augmented errors could be associated with modulation of the fronto-centro-parietal network resembling that in unstable stance situations. In addition, the right temporo-frontal and temporo-parietal connectivity at the beta band were enhanced with EA during stabilometer stance. To our knowledge, no previous studies have directly specified a state-dependent beta enhancement of the connectivity in a postural task. Beta oscillations are typically associated with attentional shifts to filter irrelevant visual representations [64], and declines in beta oscillations within the frontal, temporal, parietal, and occipital cortices are linked characteristically to the maintenance of spatial working memory performance [65]. As beta oscillations in the frontoparietal network could yield negative impacts on goal-directed memory encoding and retrieval [65, 66], we speculated that the EA-related enhanced beta connectivity signifies less attention being paid to localize amplified errors, covarying with the above-mentioned decline in theta connectivity in the right parieto-temporal networks during stabilometer stance. However, the argument still remains exploratory. Collectively, unlike the regional activity of the mid-frontal theta wave, none of the functional connectivity the that was EA dependent was significantly correlated to differences in postural variables with the use of EA during stabilometer stance (*p* > 0.05)(results were not reported in detail due to lack of significant correlations). This fact suggested that modulation of the inter-regional cortical interaction played an adjunct role in EA-related changes in postural strategy during stabilometer stance. Alternatively, EA-dependent functional connectivity worked in a synergistic (or non-linear) manner to tune the postural strategy on stabilometer stance with EA. Figure 6 summarizes EA-induced cortical reorganization (regional activity and inter-regional connectivity) and hypothetical neural mechanisms to reduce postural fluctuations on the stabiometer.

## Conclusion

For young adults, virtual amplification of motor errors in visual feedback effectively improves performance on stabilometer stance with marked shift in postural regulation strategy. The functional merit involves with reorganization of local and long-range neural networks to solve postural destabilization, considering a large scale of selective changes in regional activities and inter-regional connectivity across various EEG spectral bands. In association with smaller postural fluctuations with higher mean frequency, error amplification can enhance mid-frontal theta rhythm to monitor balance reactions and detect postural errors, which elaborate frequent corrective attempts for superior stability during stabilometer stance. In addition, error amplification potentiates occipital alpha rhythm, pertaining to reduction in visual attention on spatial navigation and target localization. The beta is hypothesized to deal with perceptual conflicts due to error amplification.

## Data Availability

Data cannot be shared as participants were informed that their data would be stored confidentially, in accordance with the rules of the local ethics committee. Code to generate the EEG metrics is available under request.

## Abbreviations

EA: Error amplification
EEG: Electroencephalography
EOG: Electrooculography
FIR: Finite impulse response
MF: Mean frequency
ND-SampEn: Normalized changes in sample entropy
PLI: Phase-lag index
RE: Real error
RMS: Root mean square
RP: Real postural sway
SampEn: Sample entropy
T: Target signal
VP: Visualized postural sway
VE: Visualized error
